# Rice and Beans

**Published:** 1883-04

**Authors:** 


### RICE AND BEANS.

HALF the people of the world live almost exclusively on rice. It contains 88 per cent. of nutriment, while roast beef contains but 26.

There are countries where the almost exclusive diet of the masses is beans; these contain 87 per cent. of nutriment.

The best and cheapest food for the dense populations of Asiatic countries, therefore, is rice; and since, from the general poverty of the people, varieties of food are out of the question, bounteous nature has given them most freely that kind which is among the most nutritious of all foods. With the rest of the world, rice is more of a side dish, and is served most frequently in the form of pudding.

We venture to affirm that when the cost, the percentage of nutriment and the wholesomeness of beans are considered, there is not in the world a single article of food that can compare with them. There is no other vegetable food that answers so well as a substitute for meats. While they have so much to recommend them in other respects, they have no rival in point of economy. A quart of beans, costing ten cents, will furnish a family of five persons with food for a day.

Much of the value of beans as food depends on the manner of cooking. It would be difficult to cook them too much. They should be first boiled until soft, and then put in a baking dish, and baked until they are brown. A little salt pork or butter, but not enough to make them taste greasy, should be put in the baking pan, and cooked with them. If beans are not thoroughly cooked, they are difficult of digestion: still there is not one hotel or restaurant in a thousand that serves them sufficiently cooked; and as a rule, it is not well to call for them in such places; but at home, when prepared under the supervision of a good cook, they make a dish that is wholesome and palatable.

---

WARM WATER, of all remedies is the one of most general application.

Cotton dipped in warm water makes the best and cleanest poultice that can be used. It is the most healing application for cuts, bruises, wounds, sores, felons and other inflammations. A very convenient way in case of felon or other painful abscess is to hold the hand for hours in water as warm as can be comfortably borne.

